# A model-based cost-utility analysis of multi-professional simulation training in obstetric emergencies

**DOI:** 10.1371/journal.pone.0249031

**Published:** 2021-03-23

**Authors:** Christopher Wai Hung Yau, Erik Lenguerrand, Steve Morris, Tim Draycott, Elena Pizzo

**Affiliations:** 1 Translational Health Sciences, University of Bristol, Bristol, United Kingdom; 2 Southmead Hospital, Bristol, United Kingdom; 3 Department of Public Health and Primary Care, University of Cambridge, Cambridge, United Kingdom; 4 Department of Applied Health Research, University College London, London, United Kingdom; London School of Economics, UNITED KINGDOM

## Abstract

**Objective:**

To determine the cost-utility of a multi-professional simulation training programme for obstetric emergencies–Practical Obstetric Multi-Professional Training (PROMPT)–with a particular focus on its impact on permanent obstetric brachial plexus injuries (OBPIs).

**Design:**

A model-based cost-utility analysis.

**Setting:**

Maternity units in England.

**Population:**

Simulated cohorts of individuals affected by permanent OBPIs.

**Methods:**

A decision tree model was developed to estimate the cost-utility of adopting annual, PROMPT training (scenario 1a) or standalone shoulder dystocia training (scenario 1b) in all maternity units in England compared to current practice, where only a proportion of English units use the training programme (scenario 2). The time horizon was 30 years and the analysis was conducted from an English National Health Service (NHS) and Personal Social Services perspective. A probabilistic sensitivity analysis was performed to account for uncertainties in the model parameters.

**Main outcome measures:**

Outcomes for the entire simulated period included the following: total costs for PROMPT or shoulder dystocia training (including costs of OBPIs), number of OBPIs averted, number of affected adult/parental/dyadic quality adjusted life years (QALYs) gained and the incremental cost per QALY gained.

**Results:**

Nationwide PROMPT or shoulder dystocia training conferred significant savings (in excess of £1 billion ($1.5 billion)) compared to current practice, resulting in cost-savings of at least £1 million ($1.5 million) per any type of QALY gained. The probabilistic sensitivity analysis demonstrated similar findings.

**Conclusion:**

In this model, national implementation of multi-professional simulation training for obstetric emergencies (or standalone shoulder dystocia training) in England appeared to both be cost-saving when evaluating their impact on permanent OBPIs.

## Introduction

Shoulder dystocia is an obstetric emergency and occurs when the anterior shoulder of a baby is impacted against the mother’s pelvic bone during birth [1]. Mismanagement of this childbirth emergency, such as excessive traction on a baby’s neck, can cause permanent injuries to the nerves in the neck (known as obstetric brachial plexus injuries (OBPIs)) resulting in long term morbidity and loss of quality of life [2]. Although permanent OBPIs are relatively rare, with incidence rates of 0.192 per 1,000 births in the UK [3] and 0.11–0.22 per 1,000 births in the US [4], these injuries are important sources of litigation and therefore impact the wider health system as well as the affected individuals and their families. In England, litigation costs related to shoulder dystocia and permanent OBPIs surpassed £103 million ($151 million) in one decade (2000–2010) [5].

Training is almost ubiquitously recommended as a strategy to improve obstetric care and outcomes [6–9]. Practical Obstetric Multi-Professional Training (PROMPT) was developed in the UK by a multidisciplinary maternity team to provide simulation training for a wide range of obstetric emergencies such as shoulder dystocia, maternal sepsis and eclampsia [10]. It consists of a ‘Course in a Box’, which contains course and trainer manuals, and adaptable training materials for local use. Hospitals wishing to implement PROMPT must first send a multi-professional team to a ‘Train the Trainers’ course before they can begin training in their maternity units. The implementation of PROMPT has been associated with clinical improvements [11–14], including the eradication of permanent OBPIs [15], and is recognised as a leading example of local multi-professional training for obstetric emergencies [8].

Economic evaluations are established methods for assessing the costs and impact of many healthcare interventions [16] but there have been few cost studies, let alone evaluations, of simulation-based training [17]. Cost-utility analyses, with outcomes expressed in quality adjusted life years (QALYs), are the recommended form of economic evaluation for National Institute for Health and Care Excellence (NICE) reviews in England [18]. NICE has set a cost-effectiveness threshold of £20,000-£30,000 ($29,000-$44,000) per QALY [19], although other factors also influence whether an intervention is recommended for use in the National Health Service (NHS) [18].

The implementation and clinical effectiveness of training programmes for obstetric emergencies has been investigated [20–22], however the cost of these training programmes has largely been ignored [23]. Information on training costs is essential at all levels of the policy and practice landscape, however data on the value of training are equally important [24, 25]. Investment in maternity training continues to be in the policy spotlight in England. The Maternity Safety Training Fund in 2016 provided each NHS Trust with up to £40,000 ($59,000) to support obstetric training [26, 27]. However, this would only have covered a proportion of the annual training costs of larger maternity units [23]. There are increasing calls for this funding to be reinstated [28] and results from economic evaluations of obstetric training may help to justify further financial support.

We conducted a cost-utility analysis of a local, multi-professional obstetric emergencies training programme (PROMPT), using published improvements in the rates of permanent OBPIs [15] as a vehicle for investigation.

## Methods

**Fig 1:**
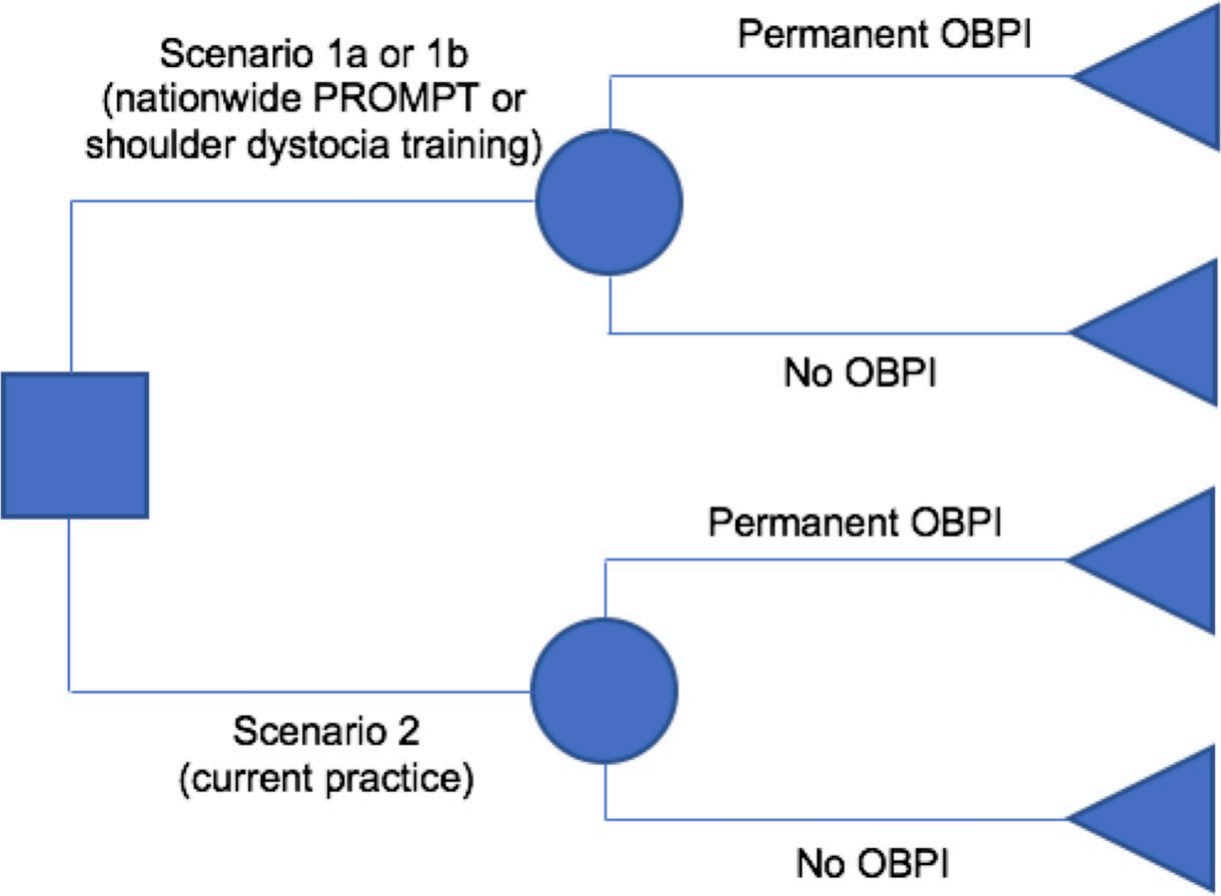
Schematic diagram of decision tree model.

A decision tree model was developed to estimate the cost-utility of adopting annual PROMPT training (scenario 1a) or just the shoulder dystocia component of PROMPT (scenario 1b) in all maternity units in England compared with current practice, where only a proportion of English units use the training programme (scenario 2) (Fig 1). Scenario 1b has been provided to demonstrate the minimum training required to influence the rate of permanent OBPIs. The training costs for scenario 1a and 1b were different but the clinical impact of both PROMPT and the standalone shoulder dystocia training was assumed to be identical, given that shoulder dystocia training is delivered as part of PROMPT.

Hypothetical cohorts of babies affected by permanent OBPIs were created for all scenarios. The analysis was conducted from the perspective of the English NHS and Personal Social Services, as per NICE guidance [18]. Personal Social Services describes additional care for vulnerable people such as those with physical disabilities. The time horizon for the model was 30 years, therefore all costs and outcomes after the first year were discounted at an annual rate of 3.5%, as recommended by NICE [18]. This time horizon was selected to allow enough time for new units to benefit from the full effect of the training (eradicating permanent OBPIs after 12 years [15]) and to ensure that the final hypothetical cohort of individuals with OBPIs were at least 18 years old.

A decision tree model was felt to be appropriate as the clinical outcome of interest (permanent OBPI) had only two health states (present or absent) and no change in health status over time. As such, all other variables were easily accounted for and adjusted to their present value using a decision tree model, despite the long time horizon.

### Ethical considerations

Ethical approval was not required for this study.

### Model inputs

The model inputs and their sources are summarised in Table 1.

**Table 1:** Summary of model inputs.

Input	Base case	Range*	Distribution	Source
Annual birth rate	648,107	611,337–671,255	Triangular	[29, 30]
**Births covered by PROMPT**				
PROMPT for 1 year	38,077	Births covered by PROMPT to follow proportions in base case during probabilistic sensitivity analysis		PROMPT data, [29]
PROMPT for 2 years	18,991			
PROMPT for 3 years	32,269			
PROMPT for 4 years	23,261			
PROMPT for 5 years	31,111			
PROMPT for 6 years	45,093			
PROMPT for 7 years	39,944			
PROMPT for 8 years	8,366			
PROMPT for 15 years	7,386			
**Probabilities of permanent OBPIs**				
Without PROMPT/ 0 years after PROMPT	0.000192	0.00019–0.0003	Beta	[3, 4]
1 year after PROMPT	0.000182	Probabilities of developing permanent OBPIs following PROMPT to follow linear assumption as per base case during probabilistic sensitivity analysis		Linear assumption
2 years after PROMPT	0.000171			
3 years after PROMPT	0.000161			
4 years after PROMPT	0.000150			
5 years after PROMPT	0.000131			
6 years after PROMPT	0.000113			
7 years after PROMPT	0.000094			
8 years after PROMPT	0.000075			
9 years after PROMPT	0.000056			
10 years after PROMPT	0.000038			
11 years after PROMPT	0.000019			
12 years after PROMPT	0.000000			
**Costs**				
Discount rate	0.035	Fixed		[18]
Cost of PROMPT per birth (first year)	£16.91	£13.53-£20.29	Triangular	[23]
	($24.79)	($19.84-$29.75)		
Cost of PROMPT per birth (subsequent years)	£15.56	£12.45-£18.67	Triangular	
	($22.82)	($18.26- $27.38)		
Cost of shoulder dystocia training per birth (first year)	£1.79 ($2.62)	£1.43-£2.14	Triangular	
		($2.10- $3.14)		
Cost of shoulder dystocia training per birth (subsequent years)	£1.43	£1.14-£1.71	Triangular	
	($2.10)	($1.67- $2.51)		
Claims inflation	0.10	Fixed		NHS Resolution
Mean total litigation cost for OBPI	£338,879	£271,103-£406,654	Triangular	
	($496,890)	($397,512- $596,267)		
**Utility scores (means)**					
Adult with OBPI	0.56	0.28–0.84	Beta	[2]
Parent of child with OBPI	0.80	0.61–0.99	Beta	
Population norm	0.86	0.62–1.00**	Beta	[31]

*Plausible ranges were generated by taking the lowest and highest birth rates in the last 10 years, using alternative baseline probabilities from an American study, discounting and inflating costs by 20% and subtracting and adding standard deviations to utility scores.

**Adding a standard deviation to the mean population utility score exceeded the upper threshold of 1.00.

#### Training coverage

The baseline birth rate in England and births by NHS Trust per model year were derived from data from 2015–2016 [29]. For convenience, the birth rate and the annual births per NHS Trust were fixed throughout the model. For scenario 2, the number of births covered by PROMPT and the years of established training were estimated using information regarding hospital/NHS Trust attendance at PROMPT ‘Train the Trainers’. Hospitals and NHS Trusts that had attended this session were assumed to be running the training programme annually thereafter. Repeat attendances were noted, but PROMPT coverage was assumed from the first ‘Train the Trainers’ day. As birthing statistics were at Trust level, only those that attended the ‘Train the Trainers’ session as Trusts could be reliably included in the estimates. However, if all hospitals within a Trust attended separately, then the births from this Trust were also considered to be covered by PROMPT after the attendance of the final hospital.

Cohorts of births covered by PROMPT were categorised by the number of years that the training had been established for as of 2015. This ranged from 1–8 years, with the exception of one NHS Trust (North Bristol) which had started training in 2000. The cohorts of births covered by PROMPT were fixed throughout the model.

#### Probabilities of permanent OBPIs

The incidence of OBPIs in the UK and Republic of Ireland was estimated at 0.40 per 1,000 vaginal births (after excluding vaginal breech and caesarean births as shoulder dystocia is less likely to occur during these deliveries) and 48% of 6-month old infants had partial or no recovery from their OBPIs, suggesting that these were permanent injuries [3]. Using this data, the incidence of permanent OBPIs in the UK was set at 0.192 per 1,000 vaginal births, or 0.192‰ (baseline probability).

This probability applied to births without PROMPT coverage and remained constant for the duration of the model. PROMPT has been associated with a reduction in permanent OBPIs [15]. After 4 years of established training (excluding the first year of implementation), the incidence of permanent OBPIs was 0.15 per 1,000 vaginal births. By the end of 12 years of established training, this had fallen to 0‰ [15]. The clinical impact of PROMPT throughout the model was set to replicate these figures, using a linear assumption to determine the probability of permanent OBPIs for each year since the implementation of PROMPT. The incidence rate of permanent OBPIs was assumed to be constant at 0‰ after 12 years of established training.

#### Creating hypothetical cohorts of babies with permanent OBPIs

Hypothetical cohorts of babies affected by permanent OBPIs were generated for all scenarios for each model year. For births not covered by PROMPT, the annual permanent OBPI rate was fixed at the baseline probability (0.192‰). The cohorts of births covered by PROMPT were combined with the appropriate, extrapolated probabilities of permanent OBPIs for each year of the model (Table 1). The impact of PROMPT and standalone shoulder dystocia training was assumed to be the same, meaning that the cohorts for scenarios 1a and 1b would be identical.

#### Cost data

PROMPT training costs were extracted from our previous micro-costing analysis. Costs for equipment, printing, administration, and room hire were included, as well as costs related to releasing maternity staff to attend training [23]. Costs were calculated in 2017 UK pounds sterling [32]. To improve comparability with other studies, costs have also been presented in 2017 US dollars [33]. Using this data and birth data from 2015–2016 [29], the cost of general training per birth was £16.91 ($24.79) in the first year and £15.56 ($22.82) in subsequent years. As shoulder dystocia training is a core component of PROMPT and is most likely to have an effect on the rate of permanent OBPIs, the cost of this training alone has been calculated to enable more targeted analyses [15, 23]. This estimation was based on fifty 30-minute sessions (allowing for ten people to attend each session) of shoulder dystocia training to train all staff in that maternity unit for one year [23]. The estimated standalone shoulder dystocia training costs can be found in the S3 File. With this approach, unit level shoulder dystocia training was estimated to cost £1.79 ($2.62) per birth in the first year and £1.43 ($2.10) per birth in subsequent years.

The cost of an OBPI was based on its mean litigation cost. NHS Resolution, the national agency that handles all medical indemnity claims against the English NHS, provided 10 years’ worth of settled claims relating to shoulder dystocia and/or OBPIs (2006/07-2016/17). In this period, there were 376 settled claims, costing a total of £127,418,372 ($186,830,458). The mean cost per OBPI claim, and therefore the mean cost of the injury, was £338,879 ($496,890). Claims Inflation for the damages component of claims was 10% per annum (which included a 2.5% personal injury discount rate) (personal communication). This was accounted for in the model.

#### Utility scores and Quality Adjusted Life Years (QALYs)

The mean utility scores for adults (aged 18 years and over) with OBPIs and parents of children with OBPIs was extracted from our previously published study [2]. The total affected adult QALYs per model year were calculated using the mean utility score for adults with OBPIs, the number of individuals with OBPIs and the number of years they would have been adults for by the end of the model’s time horizon (30 years). Similarly, the total parental QALYs per year were calculated by using the appropriate utility score, the number of individuals with OBPIs and the number of years these individuals would have been children by the end of the model. The affected adult and parental QALYs were then combined (with equal weighting) to produce a dyadic QALY. Mean utility scores (population norms) from the 2008 Health Survey for England [31] were used to determine the adult QALYs and parental QALYs when there were no OBPIs.

### Model outputs

Outcomes for the entire simulated period included the following: total costs for PROMPT training or shoulder dystocia training (including costs of OBPIs), number of OBPIs averted, number of affected adult/parental/dyadic QALYs gained and the incremental cost per QALY gained (incremental cost effectiveness ratio (ICER)).

### Probabilistic sensitivity analysis

A probabilistic sensitivity analysis was conducted to account for uncertainties in the model. The lowest and highest birth rates in England over the previous 10 years were used to create a range of births [30]. However, as before, the proportion of births covered by PROMPT remained fixed throughout. Alternative baseline probabilities for permanent OBPIs were taken from an American paper as there were no other UK studies investigating the incidence and prevalence of OBPIs– 0.19 per 1,000 vaginal births (US rate) and 0.3 per 1,000 vaginal births (rate from other countries) were used to create a plausible range of baseline probabilities [4]. This enabled the extrapolation of alternative probabilities of permanent OBPIs by individual years post PROMPT. Costs were decreased and increased by 20% to generate minimum and maximum values respectively. The discount rate and claims inflation rate were fixed. The respective standard deviations were subtracted or added to the mean adult, parental or general population utility scores to create minimum and maximum values. The ranges and assigned distributions for the parameters are summarised in Table 1. Monte Carlo simulation (1,000 simulations) was used to account for variability in the model outputs.

## Results

### Base case

The key findings for the base case are summarised in Table 2. A total 1,753 permanent OBPIs were avoided when opting for nationwide PROMPT (scenario 1a) or national shoulder dystocia training (scenario 1b) over current practice (scenario 2). Scenarios 1a and 1b also resulted in increases in adult, parental and dyadic QALYs. Both scenarios 1a and 1b conferred significant savings (in excess of £1 billion ($1.5 billion)) over scenario 2. This led to cost-savings of at least £1 million ($1.5 million) per QALY gained, irrespective of the QALY measure used. Scenarios 1a and 1b were both dominant over scenario 2, regardless of which QALY was used.

**Table 2:** Base case results.

Base case	Nationwide implementation	Scenario 2	Difference
		(Current practice)	
**OBPIs (n)**	709	2,462	-1,753
**QALYs (units)**			
Adult	44,455,206	44,454,755	451
Parental	146,867,957	146,867,240	717
Dyadic	191,323,163	191,321,995	1,168
**PROMPT (scenario 1a)**			
**Costs**	£464,530,954	£1,713,783,061	-£1,249,252,107
	($681,130,431)	($2,512,878,389)	(-$1,831,747,957)
**ICERs**			
Adult QALYs			-£2,768,111
			(-$4,058,814)
			(dominant)
Parental QALYs			-£1,742,410
			(-$2,554,853)
			(dominant)
Dyadic QALYs			-£1,069,319
			(-$1,567,916)
			(dominant)
**Shoulder dystocia training (scenario 1b)**			
**Costs**	£289,814,081	£1,648,022,191	-£1,358,208,109
	($424,947,333)	($2,416,454,826)	(-$1,991,507,491)
**ICERs**			
Adult QALYs			-£3,009,537
			(-$4,412,811)
			(dominant)
Parental QALYs			-£1,894,378
			(-$2,777,680)
			(dominant)
Dyadic QALYs			-£1,162,582
			(-$1,704,666)
			(dominant)

Figures rounded up to the nearest whole number. As a result, some of the differences may appear to have some discrepancies.

### Probabilistic Sensitivity Analysis (PSA)

Findings from the PSA are summarised in Table 3. Adopting scenario 1a or 1b over scenario 2 led to 1,733 OBPIs averted and adult QALY gains. There were parental and dyadic QALY gains too, but the confidence intervals for these were wide, indicating the uncertainty in these findings. Implementing national PROMPT (scenario 1a) or shoulder dystocia training (scenario 1b) resulted in significant cost-savings over current practice (scenario 2). Like in the base case analysis, both nationwide PROMPT and shoulder dystocia training were cost-saving (of at least £1 million ($1.5 million)) using any QALY measure in the PSA. Scenarios 1a and 1b remained dominant over scenario 2.

**Table 3:** Mean probabilistic results of PSA.

Mean probabilistic results	Nationwide implementation	Scenario 2	Difference
		(Current practice)	(95% CI)
**OBPIs (n)**	703	2,436	-1,733
			(-1,800, -1,663)
**QALYs (units)**			
Adult	48,314,960	48,314,408	553 (273, 801)
Parental	159,620,746	159,620,357	389 (-1,172, 1,727)
Dyadic	207,935,706	207,934,764	942 (-771, 2,404)
**PROMPT (scenario 1a)**			
**Mean costs**	£461,031,980	£1,696,450,297	-£1,235,418,317
			(-$1,811,463,808)
			(-£1,476,266,014, -£1,000,227,578)
	($675,999,971)	($2,487,463,779)	(-$2,164,612,924, -$1,466,609,352)
**Mean ICERs**			
Adult QALYs			-£2,426,500 (-$3,557,918) (dominant)
			(-£4,540,164, -£1,493,377)
			(-$6,657,132, -$2,189,702)
Parental QALYs			-£1,383,052 (-$2,027,935) (dominant)
			(-£27,371,470, £24,652,337)
			(-$40,134,120, $36,147,122)
Dyadic QALYs			-£2,121,318 (-$3,110,437) (dominant)
			(-£12,916,818, £5,669,881)
			(-$18,939,616, $8,313,609)
**Shoulder dystocia training (scenario 1b)**			
**Mean costs**	£287,477,566	£1,631,126,959	-£1,343,649,393 (-$1,970,160,400)
			(-£1,604,949,235, -£1,088,213,035)
	($421,521,358)	($2,391,681,758)	(-$2,353,297,999, -$1,595,620,286)
**Mean ICERs**			
Adult QALYs			-£2,639,095 (-$3,869,641) (dominant)
			(-£4,938,559, -£1,625,086)
			(-$7,241,289, -$2,382,824)
Parental QALYs			-£1,504,260 (-$2,205,660) (dominant)
			(-£29,768,033, £26,814,500)
			(-$43,648,142, $39,317,449)
Dyadic QALYs			-£2,306,803 (-$3,382,409) (dominant)
			(-£14,046,373, £6,166,821)
			(-$20,595,855, $9,042,260)

Figures rounded up to the nearest whole number. As a result, some of the differences may appear to have some discrepancies.

**Fig 2:**
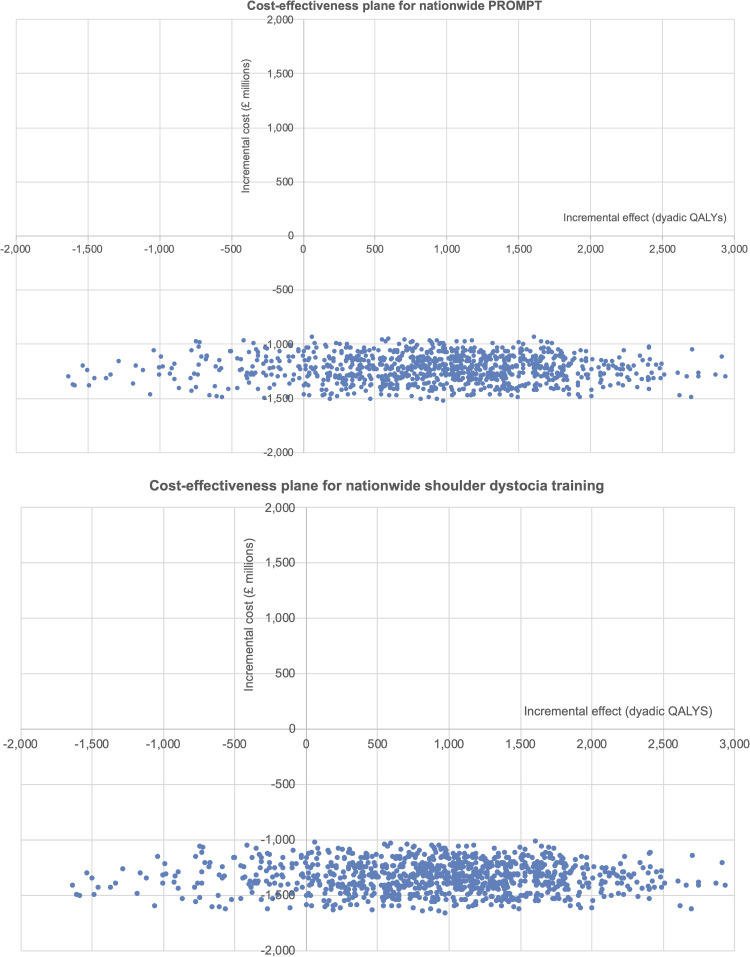
Cost-effectiveness planes for nationwide PROMPT (scenario 1a) and nationwide shoulder dystocia training (scenario 1b) using dyadic QALYs.

However, there were large confidence intervals for the ICERs when using parental or dyadic QALYs. The incremental costs for PROMPT (scenario 1a) and shoulder dystocia training (scenario 1b) and incremental QALYs (adult, parental and dyadic) of each Monte Carlo simulation have been plotted on cost-effectiveness planes. For nationwide PROMPT, the majority of the simulations lie in the bottom-right quadrant (positive effect with decreased costs) when using dyadic QALYs (see Fig 2). The same is seen in the cost-effectiveness plane for shoulder dystocia training using dyadic QALYs (Fig 2). These simulations suggest that scenarios 1a or 1b are likely to be more effective and less costly than scenario 2 (current practice).

**Fig 3:**
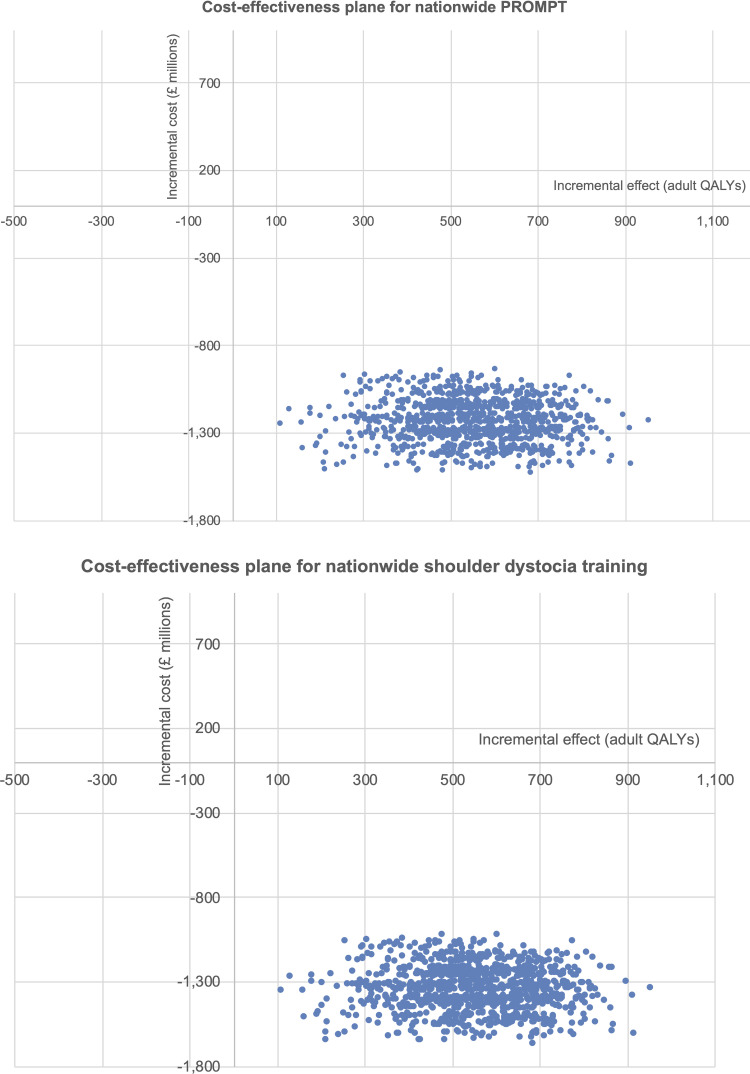
Cost-effectiveness planes for nationwide PROMPT (scenario 1a) and nationwide shoulder dystocia training (scenario 1b) using adult QALYs.

If adult QALYs are used, then all of the simulations can be found in the bottom-right quadrants for the cost-effectiveness planes for national PROMPT (scenario 1a) and shoulder dystocia training (scenario 1b) (Fig 3). These cost-effectiveness planes suggest that scenarios 1a or 1b will always be more effective and less costly than scenario 2.

**Fig 4:**
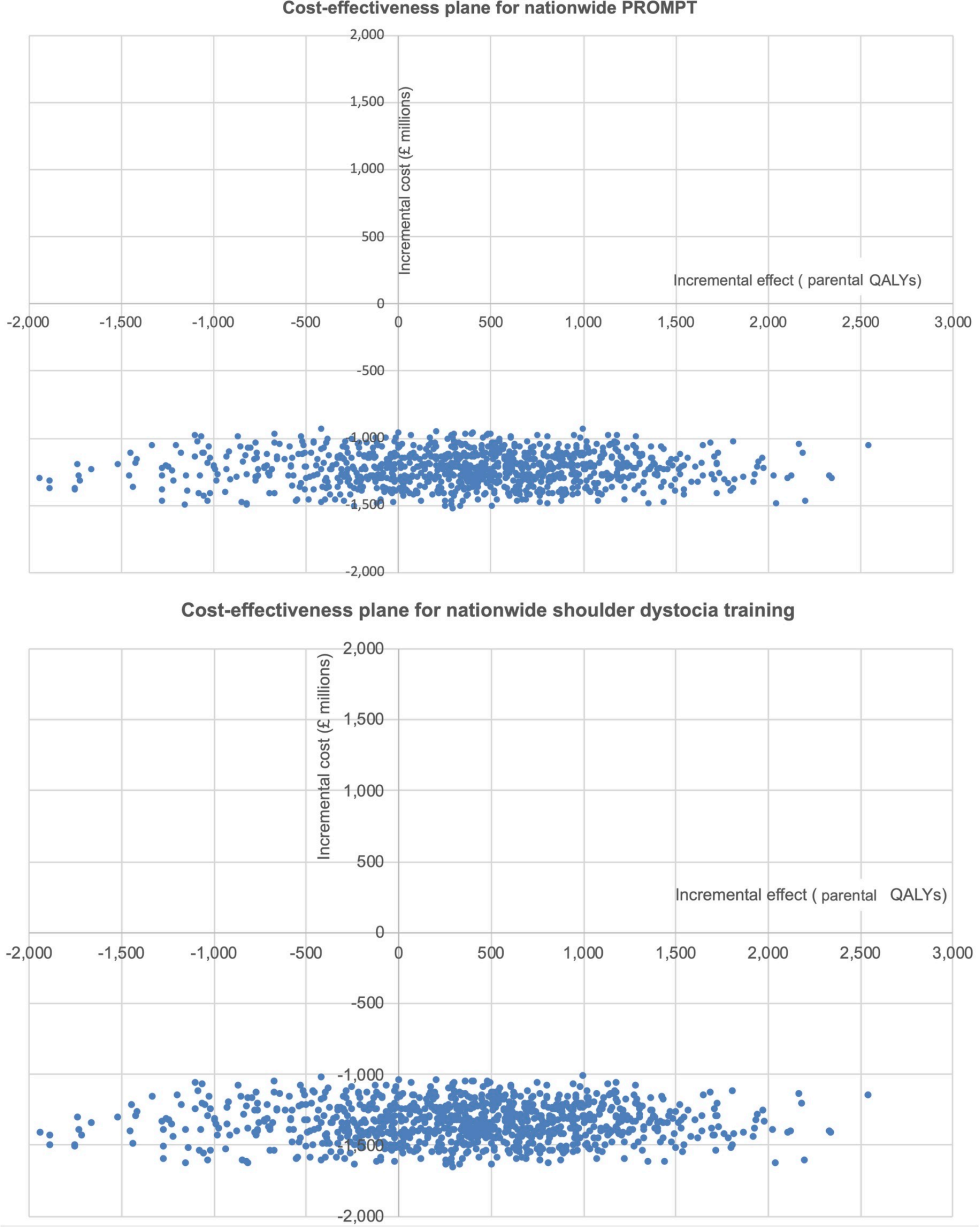
Cost-effectiveness planes for nationwide PROMPT (scenario 1a) and nationwide shoulder dystocia training (scenario 1b) using parental QALYs.

If parental QALYs are used, the cost-effectiveness planes for both nationwide PROMPT (scenario 1a) and shoulder dystocia training (scenario 1b) appear similar to those dyadic QALYs (Fig 4)

## Discussion

### Key findings

This cost-utility analysis has shown that national implementation of an established, multi-professional simulation training programme for obstetric emergencies (PROMPT) (scenario 1a) or shoulder dystocia training (scenario 1b) can both be cost-saving when considering their impact on OBPIs. The PSA provided strong evidence that opting for scenarios 1a or 1b instead of current practice could result in cost-savings of over £1 billion ($1.5 billion) and savings of over £2 million ($2.9 million) per adult QALY gained over 30 years.

### Interpretation of findings

This is the first time that a maternity training package has been economically evaluated with QALYs as the health outcome. The clinical benefits of multi-professional simulation training for obstetric emergencies can now be expressed using language familiar to policymakers. Our findings offer some evidence that investment in maternity training may lead to significant long-term cost savings and should encourage more funding in effective obstetric training programmes.

This work will contribute to the meagre collection of economic evaluations of emergency obstetric training that currently exists and provides a model for future analyses for training. A recent systematic review only identified five full economic evaluations of emergency obstetric care training and all of these were conducted in low- or middle-income countries [34]. Of these five economic evaluations, only one of them was considered a cost-utility analysis. This study used disability adjusted life years (DALYs) which were derived incorrectly and did not contain any sensitivity analyses [35]. Direct comparison with our findings is difficult due to the methodological differences and weaknesses. Whilst it is understandable that economic evaluations in this field have mostly been concentrated in low- or middle-income countries, money for healthcare is limited in all settings. Detailed economic evaluations can help guide decision-makers in making the best use of limited healthcare resources. All future studies of training for obstetric emergencies should aim to prospectively collect data for use in parallel economic evaluations.

### Strengths and limitations

All economic models are based on a number of assumptions and are dependent on the quality of the model inputs. This cost-utility analysis is no different. Firstly, the national birth rate and coverage of the training programme remained fixed throughout the duration of the model. This was a strong assumption but was made to simplify the model. The estimates of births covered by PROMPT were also subject to a degree of uncertainty. The number of births covered by the training programme may be underestimated as some hospitals (and therefore their births) were discounted due to incomplete Trust attendance. This limitation was enforced because the birth data from 2015–2016 only presented birth statistics per Trust and not individual hospitals. The underlying quality of this data has also been questioned previously [36] and the discrepancy between the birth rates reported in the Provider Level Analysis [29] and by Southmead Hospital [23] suggests possible ongoing data issues. For consistency in this economic evaluation, the birth rates in the Provider Level Analysis were used even if other information was available. The births covered by PROMPT may also be overestimated. Attendance at the ‘Train the Trainers’ course may not necessarily lead to established and/or sustained training.

Although, permanent OBPIs are defined by the presence of symptoms 12 months after birth [15], the baseline probability of permanent OBPIs was estimated using clinical information at 6 months of age as this was the only data available for the UK [3]. There is no longitudinal data to support this assumption and this is a potential limitation. However, one might expect ongoing issues if there are still residual symptoms 6 months after the injury. The probabilities of permanent OBPIs following the introduction of PROMPT were generated using the available incidence data and assuming a linear relationship. However, PROMPT is a complex intervention [37] and its clinical effect may not follow a simple linear relationship. Furthermore, although we assumed that the incidence of permanent OBPIs had fallen to 0‰ after 12 years of training, it may have been possible for this rate to have been achieved years earlier. This would have resulted in more OBPIs averted, more QALY gains and more cost-savings in our model. For this reason, our findings may be relatively conservative.

The clinical effectiveness of PROMPT in preventing OBPIs was based on one observational study [15], which remains the best available evidence to date. Any interpretation of this cost-utility analysis must also consider the limitations of using associated effects, rather than those established by causation. Throughout this economic model, the clinical impact of PROMPT was assumed to match the literature across all sites. In reality, such results may be difficult to achieve. PROMPT has been introduced in a variety of healthcare settings but similar improvements in the rate of permanent OBPIs have not yet been reported [12–14]. A recent ethnographic study of Southmead Hospital–the site of the most noteworthy clinical improvements associated with PROMPT–highlighted that the intervention (PROMPT) and its context shaped and affected each other [38]. This finding is an important reminder that the success and sustainability of PROMPT may depend on various contextual factors. A sensitivity analysis exploring the potential effect of differing training outcomes (i.e. probabilities of permanent OBPIs) would have been useful. However, there were insufficient data from the original study to formulate a plausible range of training efficacy [15]. There were also no suitable data from other PROMPT studies.

One study estimated the lifetime cost of an OBPI to be $1 million although the basis of this calculation is unclear [39]. A more robust measure of the cost is the mean litigation cost associated with the injury. The damages sum is determined by a number of elements, some of which are directly incurred by the sufferer, such as past losses, future cost of care, and adaptations to the home [40]. However, these are costs which are over and above standard NHS care, so costs associated with care provided by the NHS, such as hospital clinic appointments or surgery, have not been included in this economic evaluation. The mean litigation cost for an OBPI is at the very least a partial representation of the cost of the injury to the NHS, which pays for the entirety of successful claims. The mean litigation cost served as a useful proxy in the absence of any cost of illness data for permanent OBPIs. The recent change in the personal injury discount rate to minus 0.25% [41] was not accounted for in the model and this is likely to have led to an underestimation of the cost of claims. Rather than discounting future claims, the new rate would inflate claims costs in the future. As such, the cost of OBPIs and therefore the potential cost-savings may be greater than our findings suggest.

There is growing recognition of the effects of illnesses on family members and/or caregivers [42–44]. These effects are expected to be incorporated into economic evaluations [18] but are still frequently omitted [45]. Productivity losses as a result of caregiving are less relevant when adopting a health system perspective, but carers’ health-related costs and effects are important and should be included as health systems, such as the NHS, aim to maximise health benefit from their expenditure [45, 46]. The best way of integrating caregiver outcomes into economic evaluations remains unclear [43] but dyadic QALYs have been suggested [42, 46, 47]. In this cost-utility analysis, dyadic QALYs were created by combining the QALYs of adults with OBPIs with the QALYs of parents of children with OBPIs to illustrate the total impact of PROMPT. Child QALYs were not included as there were no suitable data in the published literature to construct them.

The uncertainties surrounding the cost of training have been discussed before [23]. One could argue that training costs are usually fixed and independent of births; however, training costs were normalised per birth in this analysis to enable training costs for a specific maternity unit to be more generalisable.

The lowest and highest birth rates in England over the previous 10 years were used to create a range of births for the PSA. Alternatively, birth rates could have been studied during the previous 30 years to identify trends which could potentially have been incorporated into the model. This may have been a more sophisticated way of modelling the annual birth rate. However, our approach was purposefully adopted to simplify an already complex model.

The PSA revealed uncertainty in the parental and dyadic QALY gains as demonstrated by the wide confidence intervals for these figures. The ICERs using parental and dyadic QALYs also had wide confidence intervals, and this uncertainty was reflected in their cost-effectiveness planes, where some of the simulations in the PSA fell within the bottom-left quadrant (less effective, less costly). This might be explained by the parental utility score being higher than the utility score for the general population for some simulations, which could be plausible. The other confidence intervals were relatively narrow, so there is reasonable evidence that national implementation of PROMPT or shoulder dystocia training could lead to avoided OBPIs, substantial cost-savings in the region of £1 billion ($1.5 billion) and significant money saved per adult QALY gained.

### Implications for policy

An economic evaluation of simulation-based training in obstetric emergencies in the Netherlands investigated the costs to prevent adverse obstetric outcomes [48]. This study found that multi-professional maternity training in a medical simulation centre could be cost-effective if repeated sessions are run locally over a year [48]. The findings in our study also support sustained training, but over a number of years. Our results strengthen the argument for continued investment in maternity training instead of one-off pump-priming payments. Only then can the full clinical benefits and cost-savings of effective training be realised. The Maternity Safety Training Fund in England was a notable example of providing isolated payments for obstetric training in 2016 [26, 27]. There is now increasing pressure for this Fund to be reinstated [28] and we hope that our findings can convince policymakers in England and around the world to continue investment in maternity training. Policymakers may also wish to improve uptake of effective training programmes associated with better clinical outcomes by making them eligible for financial support. Not all obstetric training programmes are effective [49] and some have led to worse outcomes [50]. It would therefore be vital for policymakers to help signpost clinicians and managers to the best training available.

## Conclusions

This is the first cost-utility analysis of a multi-professional, simulation-based training programme for obstetric emergencies. Nationwide implementation of PROMPT or shoulder dystocia training in England appeared to both be cost-saving when evaluating its impact on permanent OBPIs. These findings should inform policymakers’ decision-making regarding healthcare expenditure and should encourage more investment in clinically effective obstetric emergencies training.

## Supporting information

S1 File Supplementary analyses. (DOCX)

S2 File Raw data and probabilistic sensitivity analysis. (XLSX)

S3 File Estimated standalone shoulder dystocia training costs. (TIF)
