# Supplementary material for: A model-based cost-utility analysis of multi-professional simulation training in obstetric emergencies
Source: PLoS One. 2021 Mar 23;16(3):e0249031. doi: 10.1371/journal.pone.0249031 (PMC7987166; doi:10.1371/journal.pone.0249031)
Supplement: S1 File — (DOCX) [file pone.0249031.s001.docx]

#### Univariate sensitivity analyses

Instead of using the reference discount rate of 3.5%, the discount rate was set at 1.5% as suggested in NICE guidance for one univariate sensitivity analysis.

For the other univariate analyses, each cohort of births covered by PROMPT, except the births covered by PROMPT for 15 years, were reduced by 25% and 75% to account for possible suboptimal uptake of training following the ‘Train the Trainers’ sessions. North Bristol NHS Trust is the base for PROMPT and has consistently run annual training courses since 2000. These births therefore did not need to be altered in the univariate analyses.

All three univariate analyses were run independently (Table S1).

#### Multivariate analyses

Input parameters were adjusted simultaneously to create the ‘best case’ and ‘worst case’. The best case was generated by altering the following model inputs:

- Maximum baseline probability for permanent OBPIs (to increase the prevalence of the condition)
- Minimum costs for training
- Maximum litigation costs

The worst case was created by changing the following model parameters:

- Minimum baseline probability for permanent OBPIs (to decrease the prevalence of the condition)
- Maximum costs for training
- Minimum litigation costs

These two multivariate sensitivity analyses were run independently and are summarised in Table S1.

Table S1 Univariate and multivariate analyses

| **Type of sensitivity analysis** | **Model inputs adjusted** |
| --- | --- |
| Univariate analysis 1 | Discount rate set at 1.5% |
| Univariate analysis 2 | PROMPT coverage (except North Bristol births) reduced by 25% |
| Univariate analysis 3 | PROMPT coverage (except North Bristol births) reduced by 75% |
| Multivariate analysis 1 (best case) | Maximum baseline probability for permanent OBPIs, minimum training costs, maximum litigation costs |
| Multivariate analysis 2 (worst case) | Minimum baseline probability for permanent OBPIs, maximum training costs, minimum litigation costs |

#### Univariate sensitivity analyses

Running the decision model again using a discount rate of 1.5% produced similar results as the base case (Table S2). Reducing the initial PROMPT coverage by 25%, resulted in more permanent OBPIs avoided (n=2,032). However, the ICERs for scenarios 1a and 1b were very similar to those in the base case. Both scenarios 1a and 1b were dominant over scenario 2, irrespective of the QALY measure (Table S3).

Reducing the starting PROMPT coverage by 75%, resulted in even more permanent OBPIs avoided (n=2,557) but again, very comparable ICERs to the base case for scenarios 1a and 1b (Table S4). As before, both scenarios 1a and 1b were dominant over scenario 2.

#### Multivariate sensitivity analyses

The results of the best and worst cases can be found in Table S5 and Table S6. As before, OBPIs were avoided and adult, parental and dyadic QALYs were gained when adopting scenario 1a or 1b over scenario 2 in both best and worst case analyses. Opting for scenario 1a or 1b over scenario 2 resulted in significant savings of over £2.5 billion in the best case analysis, and around £1 billion in the worst case analysis. Like the base case, this meant that there were considerable cost-savings per QALY gained, regardless of the QALY measure used. Scenarios 1a and 1b remained dominant over scenario 2 in both multivariate sensitivity analyses.

Table S2 Univariate sensitivity analysis with 1.5% discount rate

| **With 1.5% discount rate** | **Nationwide implementation** | **Scenario 2**  **(Current practice)** | **Difference** |
| --- | --- | --- | --- |
| **OBPIs (n)** | 709 | 2,462 | -1,753 |
| **QALYs (units)** |  |  |  |
| Adult | 47,838,978 | 47,838,463 | 515 |
| Parental | 177,854,110 | 177,853,173 | 937 |
| Dyadic | 225,693,089 | 225,691,637 | 1,452 |
| **PROMPT**  **(scenario 1a)** |  |  |  |
| **Costs (£)** | £540,252,955 | £2,436,863,322 | -£1,896,610,367 |
| **ICERs (£)** |  |  |  |
| Adult QALYs |  |  | -£3,683,281  (dominant) |
| Parental QALYs |  |  | -£2,023,907  (dominant) |
| Dyadic QALYs |  |  | -£1,306,181  (dominant) |
| **Shoulder dystocia training (scenario 1b)** |  |  |  |
| **Costs (£)** | £316,633,148 | £2,352,653,848 | -£2,036,020,700 |
| **ICERs (£)** |  |  |  |
| Adult QALYs |  |  | -£3,954,020  (dominant) |
| Parental QALYs |  |  | -£2,172,675  (dominant) |
| Dyadic QALYs |  |  | -£1,402,192  (dominant) |
| Figures rounded up to the nearest whole number. As a result, some of the differences may appear to have some discrepancies | | | |

Table S3 Univariate sensitivity analysis with 25% reduction in initial PROMPT coverage

| **PROMPT coverage reduced by 25%** | **Nationwide implementation** | **Scenario 2**  **(Current practice)** | **Difference** |
| --- | --- | --- | --- |
| **OBPIs (n)** | 754 | 2,786 | -2,032 |
| **QALYs (units)** |  |  |  |
| Adult | 44,455,107 | 44,454,573 | 534 |
| Parental | 146,867,918 | 146,867,084 | 834 |
| Dyadic | 191,323,025 | 191,321,657 | 1,368 |
| **PROMPT (scenario 1a)** |  |  |  |
| **Costs (£)** | £482,638,481 | £1,929,984,344 | -£1,447,345,863 |
| **ICERs (£)** |  |  |  |
| Adult QALYs |  |  | -£2,709,475  (dominant) |
| Parental QALYs |  |  | -£1,735,957  (dominant) |
| Dyadic QALYs |  |  | -£1,058,059  (dominant) |
| **Shoulder dystocia training (scenario 1b)** |  |  |  |
| **Costs (£)** | £307,862,833 | £1,880,167,052 | -£1,572,304,219 |
| **ICERs (£)** |  |  |  |
| Adult QALYs |  |  | -£2,943,401  (dominant) |
| Parental QALYs |  |  | -£1,885,832  (dominant) |
| Dyadic QALYs |  |  | -£1,149,408  (dominant) |
| Figures rounded up to the nearest whole number. As a result, some of the differences may appear to have some discrepancies | | | |

Table S4 Univariate sensitivity analysis with 75% reduction in initial PROMPT coverage

| **PROMPT coverage reduced by 75%** | **Nationwide implementation** | **Scenario 2**  **(Current practice)** | **Difference** |
| --- | --- | --- | --- |
| **OBPIs (n)** | 841 | 3,398 | -2,557 |
| **QALYs (units)** |  |  |  |
| Adult | 44,454,916 | 44,454,243 | 673 |
| Parental | 146,867,842 | 146,866,793 | 1,049 |
| Dyadic | 191,322,758 | 191,321,036 | 1,722 |
| **PROMPT (scenario 1a)** |  |  |  |
| **Costs (£)** | £517,672,708 | £2,339,259,835 | -£1,821,587,127 |
| **ICERs (£)** |  |  |  |
| Adult QALYs |  |  | -£2,706,604  (dominant) |
| Parental QALYs |  |  | -£1,736,166  (dominant) |
| Dyadic QALYs |  |  | -£1,057,699  (dominant) |
| **Shoulder dystocia training (scenario 1b)** |  |  |  |
| **Costs (£)** | £342,779,508 | £2,321,329,698 | -£1,978,550,190 |
| **ICERs (£)** |  |  |  |
| Adult QALYs |  |  | -£2,939,828  (dominant) |
| Parental QALYs |  |  | -£1,885,768  (dominant) |
| Dyadic QALYs |  |  | -£1,148,839  (dominant) |
| Figures rounded up to the nearest whole number. As a result, some of the differences may appear to have some discrepancies | | | |

Table S5 Best case analysis

| **Best case** | **Nationwide implementation** | **Scenario 2**  **(Current practice)** | **Difference** |
| --- | --- | --- | --- |
| **OBPIs (n)** | 828 | 3,791 | -2,963 |
| **QALYs (units)** |  |  |  |
| Adult | 44,454,789 | 44,453,669 | 1,120 |
| Parental | 146,867,840 | 146,866,535 | 1,304 |
| Dyadic | 191,322,628 | 191,320,204 | 2,424 |
| **PROMPT (scenario 1a)** |  |  |  |
| **Costs (£)** | £530,270,086 | £3,118,254,576 | -£2,587,984,491 |
| **ICERs (£)** |  |  |  |
| Adult QALYs |  |  | -£2,310,208  (dominant) |
| Parental QALYs |  |  | -£1,984,433  (dominant) |
| Dyadic QALYs |  |  | -£1,067,482  (dominant) |
| **Shoulder dystocia training (scenario 1b)** |  |  |  |
| **Costs (£)** | £390,496,588 | £3,065,645,880 | -£2,675,149,292 |
| **ICERs (£)** |  |  |  |
| Adult QALYs |  |  | -£2,388,018  (dominant) |
| Parental QALYs |  |  | -£2,051,269  (dominant) |
| Dyadic QALYs |  |  | -£1,103,436  (dominant) |
| Figures rounded up to the nearest whole number. As a result, some of the differences may appear to have some discrepancies | | | |

Table S6 Worst case analysis

| **Worst case** | **Nationwide implementation** | **Scenario 2**  **(Current practice)** | **Difference** |
| --- | --- | --- | --- |
| **OBPIs (n)** | 708 | 2,461 | -1,753 |
| **QALYs (units)** |  |  |  |
| Adult | 44,455,209 | 44,454,758 | 451 |
| Parental | 146,867,958 | 146,867,241 | 717 |
| Dyadic | 191,323,167 | 191,321,999 | 1,168 |
| **PROMPT (scenario 1a)** |  |  |  |
| **Costs (£)** | £448,334,908 | £1,399,709,328 | -£951,374,420 |
| **ICERs (£)** |  |  |  |
| Adult QALYs |  |  | -£2,108,069  (dominant) |
| Parental QALYs |  |  | -£1,326,942  (dominant) |
| Dyadic QALYs |  |  | -£814,345  (dominant) |
| **Shoulder dystocia training (scenario 1b)** |  |  |  |
| **Costs (£)** | £238,674,661 | £1,320,796,284 | -£1,082,121,623 |
| **ICERs (£)** |  |  |  |
| Adult QALYs |  |  | -£2,397,781  (dominant) |
| Parental QALYs |  |  | -£1,509,303  (dominant) |
| Dyadic QALYs |  |  | -£926,261  (dominant) |
| Figures rounded up to the nearest whole number. As a result, some of the differences may appear to have some discrepancies | | | |
