# Supplementary figures and images for: A model-based cost-utility analysis of multi-professional simulation training in obstetric emergencies
Source: PLoS One. 2021 Mar 23;16(3):e0249031. doi: 10.1371/journal.pone.0249031 (PMC7987166; doi:10.1371/journal.pone.0249031)

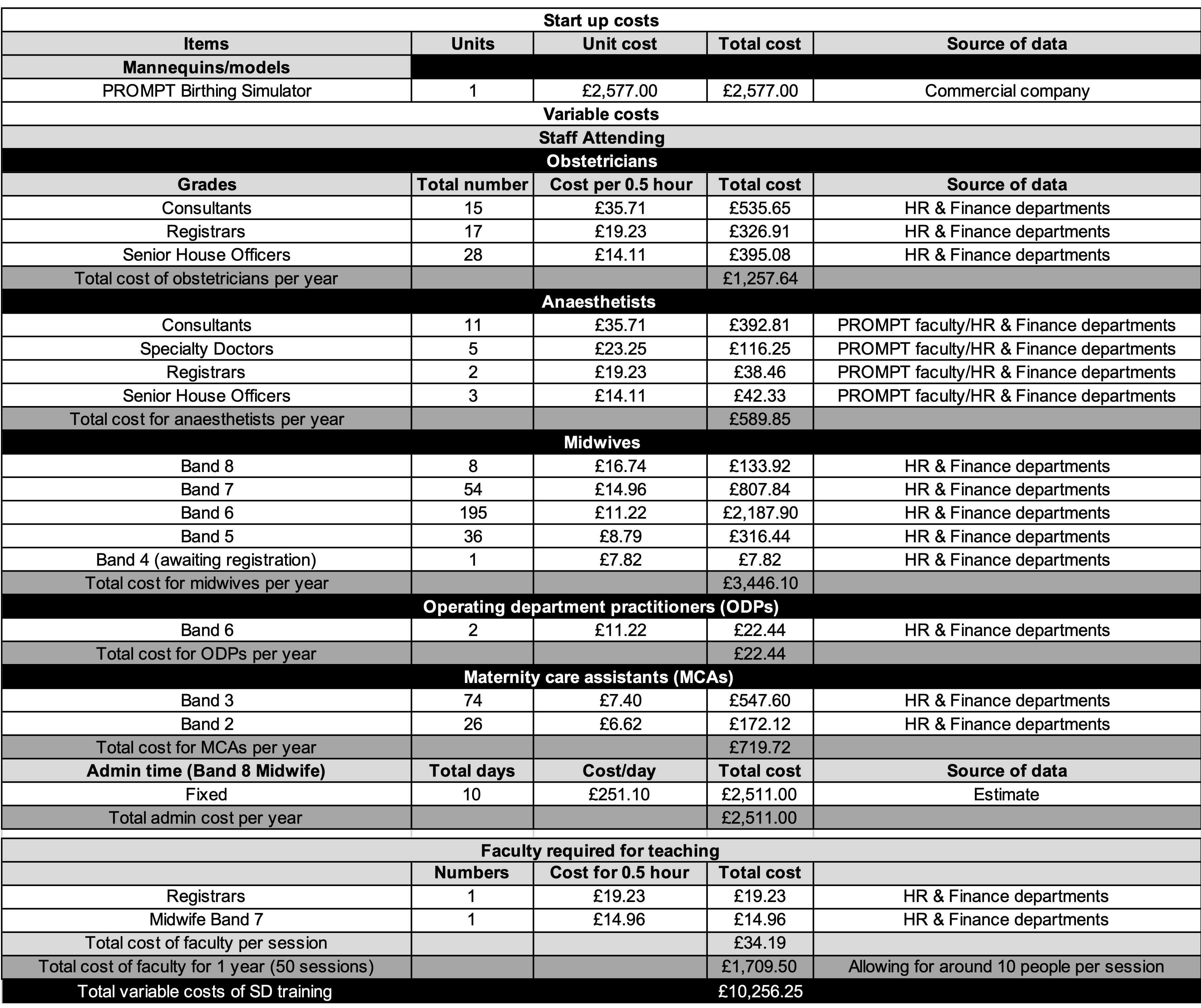

Supplement: S3 File — (TIF) [file pone.0249031.s003.tif]
